# Gamified antimicrobial decision support app (GADSA) changes antibiotics prescription behaviour in surgeons in Nigeria: a hospital-based pilot study

**DOI:** 10.1186/s13756-023-01342-9

**Published:** 2023-12-06

**Authors:** Susanne Luedtke, Caroline Wood, Olajumoke Olufemi, Patrick Okonji, Eneyi E. Kpokiri, Anwar Musah, Funmi Bammeke, Bamidele Mutiu, Rufus Ojewola, Olufemi Bankole, Adesoji Ademuyiwa, Chibuzo Ekumankama, Ayibanoah Theophilus, Neni Aworabhi-Oki, Laura Shallcross, Andreea Molnar, Sue Wiseman, Andrew Hayward, Georgiana Birjovanu, Carmen Lefevre, Stylianos Petrou, Folasade Ogunsola, Patty Kostkova

**Affiliations:** 1 UCL IRDR Centre for Digital Public Health in Emergencies, University College London, Gower Street, London, WC1E 6BT UK; 2 Department of Medical Microbiology, College of Medicine, University of Lagos/Lagos University Teaching Hospital, Lagos, Nigeria; 3 Faculty of Infectious and Tropical Diseases, London School of Hygiene and Tropical Medicine, London, UK; 4 Department of Pharmacy, Niger Delta University Teaching Hospital, Okolobiri, Bayelsa State Nigeria; 5 Department of Sociology, University of Lagos, Lagos, Nigeria; 6 Department of Medical Microbiology, Lagos State University College of Medicine/Lagos State University Teaching Hospital, Lagos, Nigeria; 7 Urology Unit, Department of Surgery, College of Medicine, University of Lagos/Lagos University Teaching Hospital, Lagos, Nigeria; 8 Neurosurgery Unit, Department of Surgery, College of Medicine, University of Lagos/Lagos University Teaching Hospital, Lagos, Nigeria; 9 Paediatric Surgery Unit, Department of Surgery, College of Medicine, University of Lagos/Lagos University Teaching Hospital, Lagos, Nigeria; 10 Department of Ophthalmology, Lagos State University Teaching Hospital, Lagos, Nigeria; 11 Department of Surgery, Niger Delta University Teaching Hospital, Okolobiri, Bayelsa State Nigeria; 12 Institute of Infectious Diseases Informatics, University College London, London, UK; 13 School of Software and Electrical Engineering, Swinburne University of Technology, Melbourne, Australia; 14 Institute of Epidemiology and Public Health, University College London, London, UK; 15 UCL Centre for Behaviour Change, University College London, London, UK

**Keywords:** Perioperative antibiotic prophylaxis, Antibiotics, Prescribing, Guideline adherence, Africa, Nigeria

## Abstract

**Aims:**

Surgical Antibiotic Prophylaxis (SAP) in Nigeria is often not evidence based. The aim of this study is to test if the GADSA application can change prescription behaviour of surgeons in Nigeria. In addition, the study aims to identify AMS strategies and policies for the future.

**Methods:**

The GADSA gamified decision support app uses WHO and Sanford prescribing guidelines to deliver real-time persuasive technology feedback to surgeons through an interactive mentor. The app can advise on whether clinician’s decisions align with SAP recommendations and provides the opportunity for clinicians to make adjustments. Twenty surgeons actively participated in a 6-month pilot study in three hospitals in Nigeria. The surgeons determined the risk of infection of a surgical procedure, and the need, type and duration of SAP. The study used a longitudinal approach to test whether the GADSA app significantly changed prescribing behaviour of participating surgeons by analysing the reported prescription decisions within the app.

**Results:**

321 SAP prescriptions were recorded. Concerning the surgical risk decision, 12% of surgeons changed their decision to be in line with guidelines after app feedback (*p* < 0.001) and 10% of surgeons changed their decision about the need for SAP (*p* = 0.0035) to align with guidelines. The change in decision making for SAP use in terms of “type” and “duration” to align with guidelines was similar with 6% and 5% respectively (both *p*-values < 0.001).

**Conclusion:**

This study suggests that the GADSA app, with its game based and feedback feature, could significantly change prescribing behaviour at the point of care in an African setting, which could help tackle the global challenge of antibiotic resistance.

## Introduction

### Antimicrobial resistance

Antimicrobial resistance (AMR) is a timely public health threat driven by the overuse of anti-infective medicine in humans, agriculture and animal husbandry as well as inadequate infection control measures [1]. The spread of AMR in Low- and Middle-income Countries (LMICs) is complex and multifactorial in nature. Inadequate laboratory facilities and inconsistent reporting of culture results to physicians can facilitate the spread of resistant bacteria [2, 3]. Additional factors known to contribute to AMR include the lack of appropriate regulatory mechanisms regarding drug quality and availability, as well as inadequate education of the population and physicians [4]. Understanding the behaviour of both patients and healthcare professionals related to the use and prescription of antibiotics (AB) is key to tackling rising rates of AMR.

### Surgical antibiotic prophylaxis and antimicrobial stewardship

Surgical antibiotic prophylaxis (SAP) is a brief course of antibiotics given immediately before or during surgery to prevent against Surgical Site Infections (SSIs). Decision-making regarding when to administer SAP is often considered a secondary task by surgeons, given the numerous urgent priorities in times of surgery [5]. The lack of priority placed on consistent and evidence-based decision making regarding SAP results in suboptimal decisions in up to 50% of surgery cases, including prescribing a broader spectrum AB than recommended or an incorrect duration [6, 7]. With only a few new antimicrobials in the clinical development pipeline [8], strategies to combat AMR need to look outside of the creation of new AB. Examples of AMR reduction activities include antimicrobial stewardship (AMS) programmes, improvements in diagnostic, stringent infection control measures, and AMR national action and policy planning. However, only 25% of Sub-Saharan African (SSA) countries have a National Action Plan (NAP) for AMR in place [9].

Education of health care workers on AMR and appropriate AB use is the first step in making a change in the AB prescribing patterns. [10] Additionally, educational measures are generally more accepted by physicians than restrictive measures [11, 12]. Therefore, AMS programmes need to ensure that the preferred behavioural change is sustainable and accepted by society and the medical environment [13]. A mobile technology provides a novel low-cost training and decision support tool for AMS, which is especially convenient for LMIC settings.

### GADSA app

An output of this study was to produce a gamified decision support smartphone application entitled Gamified Antimicrobial Stewardship Decision Support App (GADSA). GADSA creates a reinforced learning environment with game based and feedback features, incorporating little challenges and digital prizes in form of badges for on-going app usage and patient entries. Further information about gamification features can be found in the presentation by Molnar et al. and the article by Mueller et al. [14, 15]. Via a virtual mentor, the application gives feedback on the compliance of the decision regarding surgical procedure risk, antibiotic requirement, type and duration and hereby demonstrates a change towards compliance with the guidelines at the point of care.

In this paper, we present the results of a pilot study in which a group of surgeons from across three university teaching hospitals in Nigeria used the GADSA app to guide SAP decisions for elective procedures. We explored whether recommendations made by the GADSA app influenced surgeons’ decision in determining the surgical risk, requirement of SAP, type of AB and length of AB course.

The study sought to measure differences in guideline compliance between the initial decision by the surgeon, and the ultimate decision after feedback from the app.

## Materials and methods

### App creation

Various methods were applied in the app development. First, the behaviour change support system was created by using different persuasive methods, such as monitoring of app usage and direct feedback to the user. Second, the decision support system was developed based on two decision trees—verifying the surgery risk level and the prescription of surgical antibiotic prophylaxis. Finally, the main persuasive game techniques were selected and applied to the main two components of the app—behaviour change and user engagement. Further information about the vision and the development of the app can be found in the article by Wood et al. and Birjovanu et al. [16, 17].

### Participants and recruitment

Eligible participants included surgeons and pharmacists who worked closely with surgical teams. The Nigerian project PI (FO) and two research assistants (OO, PO) in Lagos recruited participants in April 2019. The project Co-PI (EK) and a senior pharmacist (AT) recruited participants in Niger Delta in July 2019. All participants were required to have access to a smartphone with an Android operating system to download the app.

### Hospital-based study area

This study was carried out in three tertiary teaching hospitals in Nigeria:

1. Lagos State University Teaching Hospital (LASUTH), initially established in 1955 as a General Hospital and subsequently converted to a tertiary care University Teaching Hospital in 2001 (http://www.lasuth.org.ng/hospital_review.html).
2. Lagos University Teaching Hospital (LUTH) enacted in 1961, it started as a 330-bed hospital and transformed into the largest teaching hospital of Nigeria with 761 beds (https://www.luth.org.ng/show/history). Both hospitals have weekly theatre activities include General, Gynaecological, Paediatric, Plastics & Reconstructive, Urological, Orthopaedic, ENT, Maxillofacial, Cardiothoracic (LUTH) and Neurosurgery, as well as Ophthalmology surgery. The two hospitals are both located in Lagos, Nigeria and are about 10 kms apart from each other.
3. Niger Delta University Teaching Hospital (NDUTH). It is the only tertiary care teaching hospital in the Niger Delta region of the Bayelsa state with 192 beds. Built in 1982 as a General Hospital, it was elevated to a teaching hospital in 2007, to serve as a training facility to the College of Health Sciences Niger Delta University. Given its significantly smaller size, the surgical load is much smaller, covering mainly general, Urology, Paediatric, Cardiothoracic, Gynaecological, Ophthalmology and Orthopaedic surgery (https://www.nduth.org.ng/about/).

### Study design

#### Pre-pilot

In all three hospitals, paper prescriptions for SAP is given to patients to collect ABs prior to their surgery from the hospital-based pharmacy. Prior to the start of the pilot, pharmacists at the three hospitals collated information about the type of SAP prescribed by surgeons over a 2-month period in their respective institutions. This information provided a baseline indication of SAP prescription prior to intervention. Surgeons were not aware of the pre-intervention collection of data.

#### Pilot: use of the GADSA smartphone application

This interventional study used a longitudinal approach to test whether the GADSA app significantly changed prescribing behaviour of participating surgeons by analysing the reported prescription decisions within the app. The pilot took place from June 1st to December 31^st^ 2019. Participating surgeons installed the app and were asked to report each SAP decisions being made throughout the period. Additionally, surgeons were invited to join a WhatsApp group, to receive support and encouragement to use the app. Further information about use of the WhatsApp groups and other pilot engagement techniques can be found in the abstract presentation by Molnar et al. [14].

**Fig. 1:**
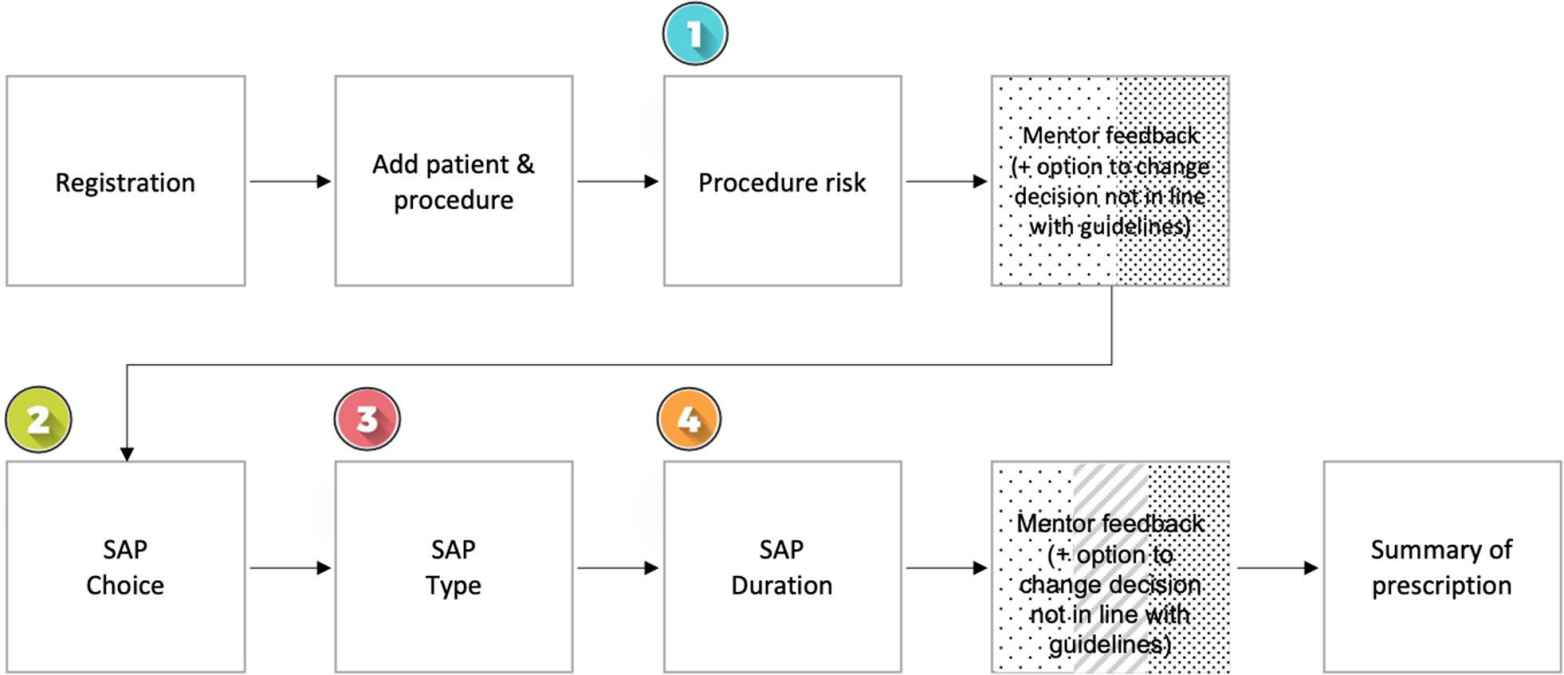
Illustrates the stages the surgeon goes through when entering data into the GADSA app and the four decisions they are asked to make. Feedback is provided after decision 1 (for procedure risk) and after decision 4 (for SAP choice, type and duration). The “Mentor feedback” box was coloured red, yellow or green (negative, neutral and positive) when providing feedback to the surgeon based on their alignment with guidelines. The summary page provides an overview of the decision and brings back the guidelines recommendations to reinforce learning

For each patient, participants had to enter the patient’s information and the procedure. Participants then made a series of decisions about the patients SAP prescription (Fig. 1).

Participants recorded:

- Whether the surgical procedure was considered low or high risk
- Whether SAP prescription was required. This was classified as “Yes” or “No” (‘SAP choice’)
- If SAP was required, the name of the SAP prescribed (‘SAP type’)
- The course duration of the SAP prescribed (‘SAP duration’)

**Fig. 2:**
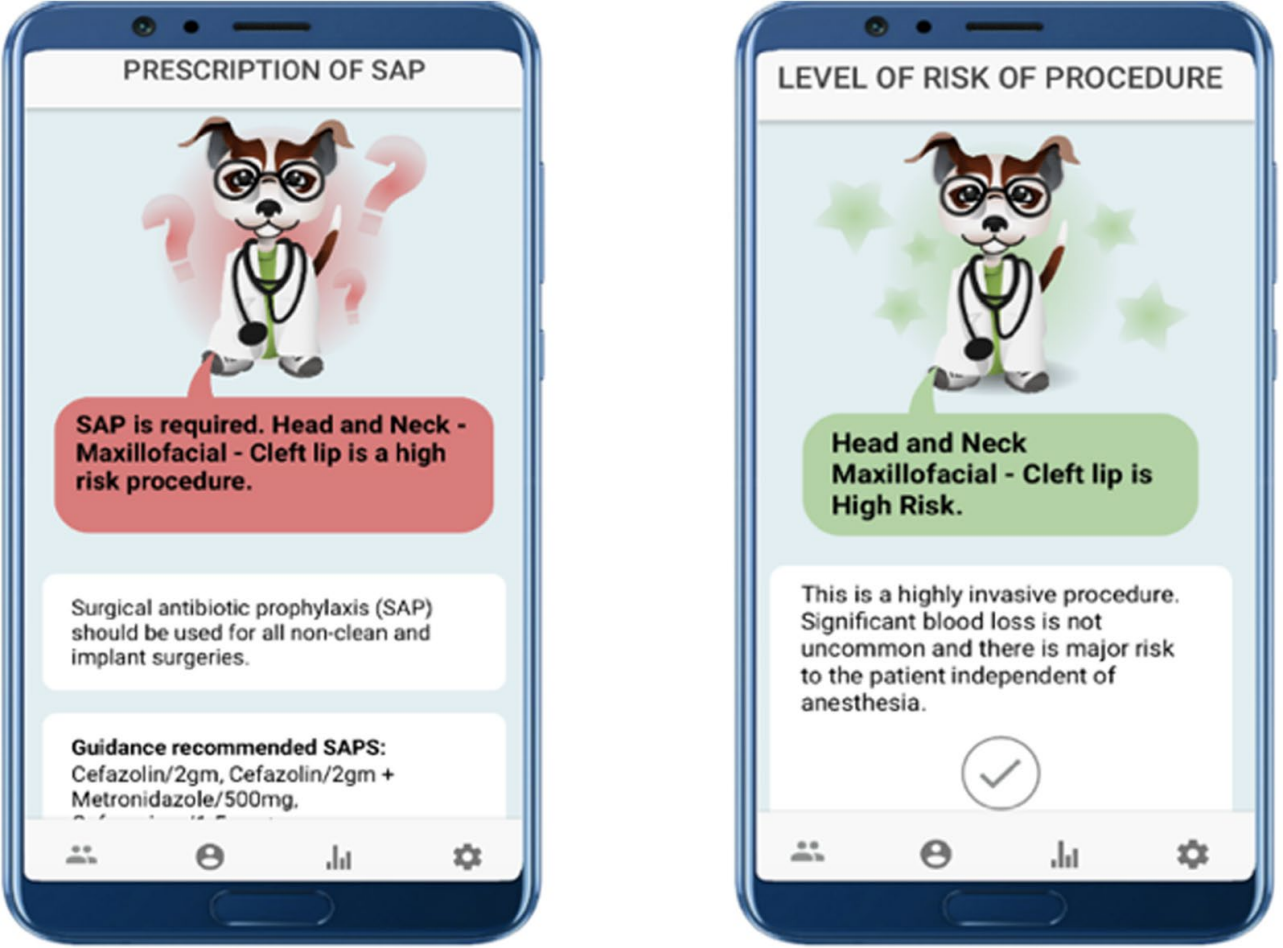
Screenshot of in-app feedback

The app integrated evidence-based prescribing guidelines from WHO and Sanford [18, 19] and delivers real-time persuasive feedback to surgeons via an interactive mentor. The virtual mentor advises the participant as to whether their pre-surgery SAP decisions aligns with standard recommendations. All technical development of the application and engagement strategies of surgeons are described in full details in the article by Birjovanu et al. [17]. To reinforce learning, when participant decisions matched the existing guideline recommendations the app provided positive feedback as well as a reiteration of the guidelines. In cases where decisions did not align with guidelines, the virtual mentor highlighted the recommended SAP prescription (Fig. 2). In cases where the guidelines did not provide a definitive recommendation–a neutral feedback was given.

**Fig. 3:**
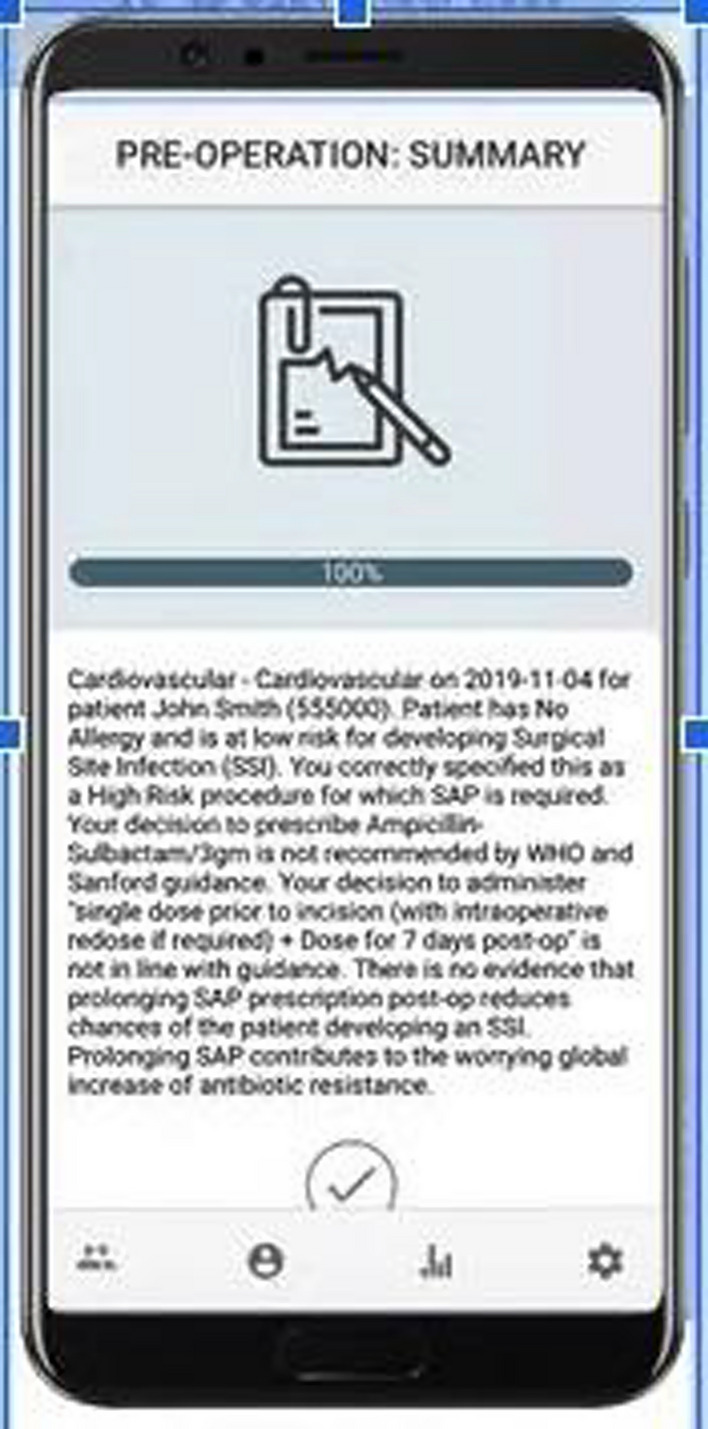
Screenshot of in-app summary page

If participant’s decisions did not align with guidelines, the app gave surgeons the opportunity to change their decisions, following provided feedback. In addition, participants were prompted to indicate their reasons for not aligning with the recommendations should they have chosen not to change their decision. In order to reinforce learning, the virtual mentor provides a summary of the decision in form of a brief overview of the case and references to guidelines (Fig. 3).

To thank participants for their involvement, all received a token to cover mobile data expenses.

Ethical approval was obtained for all phases of this study prior to data being collected from the UCL Ethics Committee (ID: 11491/001), as well as from all three Nigerian partner hospitals. The purpose for each phase of the study was described to participants and all provided consent before taking part.

### Statistical analysis

The primary outcomes were the pre- and post-decision data (i.e. initial and after feedback decision) that were recorded in the app during the study period. Changes to decision-making patterns for prescribing SAPs were measured. The surgeons documented their decisions using the decision support app longitudinally. The surgeon provided an initial, preoperative decision about the surgery risk, whether SAP will be given or not (i.e. “choice”), the SAP that will be given (i.e. “type”) and the length of prescription (i.e. “duration”) (Fig. 1). This first set of decisions are recorded within the app. Standardized feedback is then given to the user by the virtual mentor. Our hypothesis was that this feedback would influence the user to either retain their decision or to change it. This second set, whether the user retains or changes their decisions, represents their post-feedback decision. The structure of the data is therefore a ‘before and after’ format that does not assume any specific distribution. We generated 2-by-2 contingency tables to show whether a surgeon’s initial and after-feedback decisions for surgery risk and for SAP use aligned with the guidelines. To capture the changes in the decisions, we first created the following notations; C for when decisions were in concurrence with the guidelines, and N for surgeons’ decisions not aligned with the guideline. We then paired the notations as the four possible initial and after feedback responses (i.e. CC, NC, CN and NN). Possible outcomes included the four following pairings:

- CC (both decisions (initial and after feedback) were retained and were within the guideline);
- NC (the initial decision was not within guideline, and then changed (after feedback) to be within the guideline);
- CN this combination was not possible, as the app did not allow for a correct decision to be changed to an incorrect decision. It is therefore reported as 0;
- NN (both decisions (initial and after feedback) were retained and were not within the guideline);

A series of McNemar’s exact chi-squared tests were used to test the significance on the different outcomes (i.e. surgery risk, SAP use (choice, type and duration)) to see whether or not there were any significant differences in the probabilities between the discordant paired responses (i.e. NC versus CN) and whether the decisions were aligned with the WHO guidance. All tests were deemed statistically significant if the p-value was less than 0.05. Data was exported from the GADSA app as a JSON (JavaScript Object Notation) format and undergone extensive cleaning and formatting using R (version 1.2.1335) and IBM SPSS Statistics (version 26.0). All statistical analyses were conducted in R (version 1.2.1335 and 4.1.2).

## Results

### Participant demographics

65 physicians were involved in the situation analysis survey which assessed the barriers and enablers of antibiotic prescribing practices [20]. Twenty surgeons from across three different hospitals participated in the pilot. 85% of surgeons were between 25 and 44 years old, with 55% being male and 50% being junior residents. The Obstetrics and Gynaecology (O&G) as well as General Surgery departments had the highest number of participant surgeons, while the Ophthalmology department and one physician in General Surgery logged in the most patients (Table 1).

**Table 1:** A breakdown summary (by participating 3 hospitals) of the descriptive demographics of physicians using the GADSA app

Hospital	LASUTH	LUTH	NDUTH	Total
Surgeons (n)	6	8	6	20
Characteristics	N	N	N	N
Age group				
25–34	2	1	5	8
35–44	4	4	1	9
45–54	0	2	0	2
55–64	0	1	0	1
Gender				
Male	2	5	4	11
Female	4	3	2	9
Department				
Ophthalmology	3	0	0	3
O&G	0	2	4	6
Gen. Surgery	1	3	1	5
Dentistry	0	2	0	2
Paediatrics	0	1	0	1
OMF	1	0	0	1
Pharmacy	0	0	1	1
ENT	1	0	0	1
Seniority				
Junior resident	4	1	5	10
Senior resident	2	3	1	6
Consultant	0	4	0	4

Lagos State University College of Medicine (LASUTH); Lagos University Teaching Hospital (LUTH); Niger Delta University Teaching Hospital (NDUTH), Ear Nose Throat (ENT); Obstetrics & Gynaecology (O&G); Oral & Maxillofacial Surgery (OMS)

### Data collected before start of the app

Table 2 shows an overview, as well as a more detailed breakdown, of what kind of prescriptions were commonly used before the start of the app. This data was collected retrospectively by the pharmacy departments of the three hospitals across a 2-month period directly preceding the pilot.

**Table 2:** Exploring the distribution of the Pre-intervention SAP prescription data, breakdown by hospital (N = 373)

Hospitals	LASUTH	LUTH	NDUTH	Total
Antibiotic combination	N (%)	N (%)	N (%)	N (%)
AB combination covering gram-pos, gram-neg and anaerobs*	64 (45.7)	110 (59.8)	41 (83.7)	215 (57.6)
Fluoroquinolone	5 (3.6)	0	0	5 (1.3)
2nd gen. Cephalosporin	4 (2.9)	2 (1.1)	1 (2.0)	7 (1.9)
3rd gen. Cephalosporin	21 (15.0)	49 (26.6)	3 (6.1)	73 (19.6)
3rd gen. Cephalosporin + Aminoglycoside	5 (3.6)	10 (5.4)	0 (0.0)	15 (4.0)
3rd gen Cephalosporin + Metronidazole + Aminoglycoside	0	0	2 (4.1)	2 (0.5)
Metronidazole	6 (4.3)	0	2 (4.1)	8 (2.1)
Amoxicillin/clavulanic acid + Metronidazole	12 (8.6)	0	0	12 (3.2)
Meropenem	3 (2.1)	0	0	3 (0.8)
Other	20 (14)	13 (7.1)	0	33 (8.8)
Total	140	184	49	373

Lagos State University College of Medicine (LASUTH); Lagos University Teaching Hospital (LUTH); Niger Delta University Teaching Hospital (NDUTH)

*Beta-lactam Antibiotic + Metronidazole or Beta-lactamase inhibitor; Fluoroquinolone + Metronidazole

### Data collected after the start of the app

321 surgeries were entered into the app. This means a single surgeon averaged 8 surgeries during the data collection period, with a range of 1–87 surgeries. The application provided recommendations to the surgeons after the surgeon’s initial choice to either to proceed with their choice or to reconsider it.

The first decision made by surgeons was to classify the surgical procedure as low risk or high risk. The assessment of the surgery risk changed with the use of the app (Table 3). 12% of surgeons were changing their decision to be in-line with the recommended guideline after receiving feedback from the app, compared to 5% who decided not to change their decision after feedback. According to their feedback given, most of them were following the advice of a more senior clinician or from previous experience with the procedure.

**Table 3:** Summary breakdown of behaviour change and compliance to recommendation for initial and after feedback response, with results from Exact McNemar’s test

Initial & Feedback responses (Choice within guideline = C, Choice outside guideline = N)					McNemar’s test
	CC	CN	NC	NN	
Surgery Risk	268 (83%)	0 (0%)	37 (12%)	16 (5%)	*P* < 0.0001
SAP Choice	286 (89%)	0 (0%)	33 (10%)	2 (1%)	*P* = 0.0035 < 0.005
SAP Type	43 (13%)	0 (0%)	18 (6%)	260 (81%)	*P* < 0.0001
SAP Duration	41 (13%)	0 (0%)	16 (5%)	264 (82%)	*P* < 0.0001

The second decision was to determine if SAP was required (SAP choice), as some surgeries do not meet criteria for administrations of any SAP. The assessment for SAP choice showed a significant change (Table 3). Here, most of the surgeons picked the correct decision before and after feedback (i.e. CC = 286 (89%)) as expected since this question followed the previous one (i.e. if the surgery risk is high, an SAP is prescribed and vice versa). Only 2 doctors chose not to follow the guidelines. In both cases the patient risk during the surgery was reported to be high.

The third decision was the type of AB used as SAP (SAP type) chosen by the surgeon. The assessment for SAP type showed a significant change (Table 3). In 43 surgeries, the initial SAP decision aligned with the recommended guidelines, while surgeons changed their decision to be in-line with the recommended guidelines after receiving feedback in 18 surgeries. In 260 surgeries, surgeons did not change their decision to align with guidelines. A 1st generation Cephalosporin was recommended by the app in 86 surgeries (see Table 4) but was only prescribed in 4 surgeries total. A combination of AB (Beta-Lactam AB + Metronidazole or Ampicillin Sulbactam) covering gram-positive, gram-negative as well as anaerobic bacteria was recommended by the app in 57 surgeries. After receiving feedback however, out of the 154 surgeries in which this combination was initial prescribed, it was only changed in 15 surgeries to a different SAP.

**Table 4:** Surgeons initial SAP type decision, App recommended type (in accordance to WHO/ Sanford guidance) and surgeons final SAP type decision during the intervention project (N = 321)

SAP combination	Initial N (%)	App N (%)	Final N (%)
No SAP	5 (1.5)	132 (41.1)	35 (10.9))
AB combination covering gram-pos, gram-neg and anaerobs*^1^	154 (48)	57 (17.8)	139 (43.3)
Fluoroquinolone	112 (34.9)	3 (0.9)	102 (31.8)
1st gen Cephalosporin	1 (0.3)	86 (26.8)	4 (1.2)
2nd & 3rd gen Cephalosporin	24 (7.5)	4 (1.2)	19 (5.9)
Clindamycin + Gentamicin or Ciprofloxacin or Aztreonam	1 (0.3)	4 (1.2)	1 (0.3)
Fluoroquinolone or Chloramphenicol ophthalmic solution	14 (4.4)	0	14 (4.4)
Doxycycline	0	5 (1.6)	0
No recommendation recorded*^2^		14 (4.4)	
Other	10 (3.1)	16 (5)	7 (2.2)
Total	321	321	321

*^1^Beta-lactam Antibiotic + Metronidazole or Beta-lactamase inhibitor; Fluoroquinolone + Metronidazole

*^2^Includes following recommendations from the app: “No guidance available for Beta-Lactam allergic patients.”, “No guidance for patients undergoing a specific procedure available.”

The final decision (Sap Duration) follows directly from the SAP Type and as we can see from the results in Table 3, SAP Type and SAP Duration choices are almost identical. The result showed a significant change (Table 3), with a change to a correct AB duration in 16 surgeries. In the majority, AB were prescribed for 5 days after surgery (Table 5). The small discrepancies between the slightly higher incorrect choices for SAP Duration and SAP Type (i.e. NN = 260 for SAP Type and NN = 264 for SAP Duration) is because sometimes the doctors change the duration for the SAP prescription as the patient was at high risk for developing SSI.

**Table 5:** SAP duration decision made by surgeons

Decision	Initial	After feedback
SAP duration	N (%)	N (%)
No SAP given	25 (7.8)	58(18.1)
Dose up to 24 h post op	31(9.7)	31 (9.7)
Dose for 2 days post-op	19 (5.9)	18 (5.6)
Dose for 3 days post-op	30 (9.3)	19 (5.9)
Dose for 4 days post-op	22 (6.9)	11 (3.4)
Dose for 5 days post-op	156 (48.6)	150(46.7)
Dose for 7 (or more) days post-op	34(10.6)	31 (9.7)
Dose 200 mg immediately post-op	4(1.2)	3(0.9)
Total	321	321

## Discussion

### Gamified app helps AB guideline compliance

Our study results show a significant shift in guideline-concurrent AB type after receiving guidance from the virtual mentor. This represents a move forward in terms of responsible AB prescribing, as guidelines encourage the practice of prescribing narrower spectrum SAPs, which contributes less to AMR.

The results of this limited 3-hospital pilot suggest that the use of a mobile phone app can have an impact on the SAP prescribing patterns of busy surgeons. In our study we showed the potential for an app to significantly change prescription behaviours of surgeons, including shortening the recommended course-length, and becoming more compliant with standard guidelines.

Our results show the power of relatively simple AMS and educational tools, similar to a recent Cochrane review [21], in which the implementation of an antimicrobial stewardship programme was linked to a decrease in the length of the antimicrobial course.

However, the implementation of AMS programmes can be challenging. Especially in resource-limited settings like our study sites in Nigeria. There is a need to adapted AB prescribing guidelines to the local antimicrobial resistance profile; however, these data are often lacking [22]. By focusing on key interventions like decreasing the length of the AB course or working towards the elimination of using AB with overlapping spectra substantial effects can be made in resource-limited facilities with few to no infectious diseases specialists [23, 24].

### Understanding the drivers of AB overprescribing

In a recent systematic review form the WHO about overuse of medications in LMICs, it was estimated that the overuse of AB ranged between 18.4 and 97.0% [23].

In our pilot, 25% of AB were prescribed without clear indication. There are many reasons for prescribing antibiotics against standardized guidelines [20]. There is a lack of oversight, as antimicrobial stewardship programmes in Nigerian hospitals are inadequate [26] and if present, guidelines are being weakly enforced [27]. Additionally, there are factors such as cost and availability [22], social pressures from patients and senior colleagues, and a low inclination from physicians to follow policies [28].

Antibiotic overuse is driven by multiple factors, not limited to doctors prescribing without indications. We can also look to the use of overly broad or redundant combinations of multiple ABs. Unnecessary AB combinations, often used for gram positive and anaerobic bacteria, account for a large portion of AB use [29]. In our pilot project surgical patients were prescribed ABs for SAP which were too broad in their coverage. A 1st generation Cephalosporin provides a narrow spectrum and targeted use for common skin bacteria [18] and represents a safe choice in regard to efficacy, risk of side effects and risk of developing resistance [30–32]. In our pilot we found that though 86 (25%) surgeries required only a 1st generation Cephalosporin, even after feedback its singular usage was a rare choice by the surgeons.

There is no singular reason for overprescribing; one can divide the myriad reasons into individual level drivers which are clinician or patient driven, and system-level drivers which are institutions and organizations driven. Examples of individual level drivers include patient wishes and physician knowledge about AB use as well as the local resistance patterns. System based drivers include staffing, resources, and national and local guidelines [25]. In 42% of cases, surgeons in our pilot reported ‘following local practices’ as the reason for guidance discordance, while ‘patient’s environment’ requires prolonged dose in 21% of cases [20]. In both cases, the listed reasons for guideline discordance included both individual and system level-drivers.

### Workforce development and training

There are two distinct moments that are critical for training the health workforce to prescribe antibiotics responsibly: during training in medical school, and during continuing-education training for working professionals. There are training gaps in both instances, and they need to be considered separately in order to be adequately addressed. Based on the surgery risk, type and duration results of our study, the GADSA app is a prime example of an intervention that could be best suited to the iterative, daily needs of a working professional.

As part of surgeon training, sensitivity towards patient environment needs to be taught, and inclusion of this element needs to be included on a systemic (curricular) level. For example, surgeons need to be trained to consider patients living in remote area with no possibility of post-surgery follow-up. Surgeons who are aware of patient constraints are able to use existing guidelines and better tailor the AB course to ensure compliance and suitability.

### Strengths and limitations

One major strength of the app is the inclusion of prescribing surgeons and pharmacists in the design and decision-making process of the app. Taking local prescribing patterns and habits into account, will likely enhance adherence to new guidelines and empower physicians in their knowledge of following standardized guidelines and preserving their patients individual safety [23].

Another strength of the app is the unique, gamified approach to AMS. We were able to engage busy surgeons by incorporating little challenges and digital prizes [16, 17]. With a mobile-decision support tool like GADSA, health care professionals in resource-limited settings have ready access to up-to-date information via their smart phones, that is easily adaptable to local settings and in addition functions as an educational tool [33].

Limitations of the study include the researcher’s access to sensitive prescribing records, a lack of complete, electronic records, and the ability to ground—truth the existing records with patient reality. A lack of electronic medical record system makes information sharing a challenge. Pre-intervention paper data about AB used was collected by a pharmacist and transcribed into a digital format. As such, only prescriptions filled pre-surgery by the patient in the hospital pharmacy were collected. We were therefore unable to compare the data collected, pre-intervention by hand and post-intervention through the app.

Post-intervention, we are also not able to ascertain, that surgeons actually prescribed their recorded AB, instead of reporting a socially acceptable answer in the app but prescribing something else in reality. However, as we had maintained a close relationship and trust with the surgeons via the WhatsApp groups and the local PIs, this self-reporting bias was most probably minimized.

While the WhatsApp group was not designed to change behaviour and is not part of the GADSA app, one cannot exclude that the surgeons were influenced in their activities and behaviour through interaction with each other and the researchers. In a scoping review of the role of WhatsApp in medical education, Coleman and O’Connor found, that the use of WhatsApp may be effective as a learning tool [34]. Given our WhatsApp group was not designed with an educational programme and no clinical cases were discussed, it certainly helped retain and motivate surgeons to use the app, the influence on their prescribing behaviour however was likely small. An implementation of WhatsApp as an integral part of the GADSA App is a possibility for future research.

Given the lack of antimicrobial resistance data of the hospitals, the guidelines used were internationally standardized and recognized guidelines [18, 19]. With locally adapted guidelines, surgeons might have a stronger trust and therefore conviction to follow these guidelines.

Furthermore, we could not rule out that in some cases the junior surgeons entered the data on behalf of the senior surgeons. Thus, in the next development, a multi-user version will be designed to address this challenge.

## Conclusion

Antimicrobial Resistance needs to be combatted on an individual and systematic level. This study seeks to determine whether a gamified app for surgeons can promote AB stewardship and encourage responsible prescribing in order to address over prescription of ABs.

The GADSA app is a bespoke gamified app which was tailored to community needs to provide gold-standard feedback to surgeons at the front-line of AMR. Given surgeons are extremely busy, new information and educational activities need to be seamlessly included into their daily work schedule. By receiving feedback from the app, surgeons can simultaneously receive educational information about current guidelines and are prompted towards gold-standard practices, including recommended SAP.

In this small but encouraging pilot study, the GADSA app showed that feedback from the app prompted surgeons to adjust their prescribing decision to become compliant with best-practice guidelines and suggests that that this approach may be useful.

Though further holistic support and resource allocation is needed across infection control programmes, an app such as GADSA presents a first step to facilitate positive change in a low resource setting.

## Data Availability

The data are held on a secure server. In line with project policy and data privacy reasons it cannot be shared with a third party.
